# A Case of Cervical Lymphadenopathy After Vaccination Against COVID-19

**DOI:** 10.7759/cureus.15050

**Published:** 2021-05-16

**Authors:** Florinda Cardoso, Alcinda Reis, Catarina Osório, Horácio Scigliano, Mário Nora

**Affiliations:** 1 General Surgery, Centro Hospitalar de Entre Douro e Vouga, Santa Maria da Feira, PRT; 2 Radiology, Centro Hospitalar de Entre Douro e Vouga, Santa Maria da Feira, PRT; 3 Pathology, Centro Hospitalar de Entre Douro e Vouga, Santa Maria da Feira, PRT

**Keywords:** ultrasound, lymphadenopathy, axillary lymphadenopathy, cervical lymphadenopathy, covid-19 vaccination

## Abstract

The coronavirus disease 2019 (COVID-19) pandemic has caused a major global healthcare crisis, and the fields of science and medicine have been engaged in a massive effort to control and prevent the resultant deaths and morbidity. Researchers and pharmaceutical companies have developed in record time vaccines against COVID-19 that are intended to be safe and effective; however, the short validation time has been a challenge for doctors and epidemiologists, especially in light of the increase in reports emerging from various parts of the world about the adverse effects of the new vaccines.

Portugal's national regulatory authority, the National Authority of Medicines and Health Products (INFARMED), has recently granted approval for Pfizer-BioNTech (Pfizer Inc., New York, NY; BioNTech SE, Mainz, Germany) and Moderna (Moderna, Inc, Cambridge, MA) COVID-19 vaccines, and they are being rolled out to be administered among the general population. In light of this, it is important for breast surgeons, family doctors, hematologists, and radiologists to consider the effects of recent COVID-19 vaccination history as a possible cause in the differential diagnosis for patients with unilateral cervical adenopathy. The objective of this report is to present a case that involves an adverse reaction involving acute-onset cervical lymphadenopathy in a female patient that coincided with her vaccination against COVID-19, even though cervical lymphadenopathy had not been previously reported as a potential side effect of the COVID-19 vaccination.

We discuss the case of a Portuguese physician with a family history of breast cancer, who developed right cervical lymphadenopathy after receiving the first dose of the COVID-19 vaccine. Lymph node growth and ultrasound changes observed in the patient over the weeks, and a lack of information on the COVID-19 vaccine's adverse effects, prompted an in-depth study to understand its etiology.

## Introduction

With the rollout of the vaccines against coronavirus disease 2019 (COVID-19), various adverse events are being reported from different parts of the world. The most commonly reported side effects of the COVID-19 vaccines include pain in the injection site, fatigue, headache, fever, chills, and muscle and joint pain.

The presence of cervical lymphadenopathy is always a cause for anxiety for the patient and the attending physician, and the study of its etiology entails imaging and analytical examinations. Differential diagnoses regarding the cause of cervical lymphadenopathy generally take into account infections, carcinoma, or response to recent vaccinations, as has been reported in cases of vaccinations against Bacillus Calmette-Guerin (BCG), human papillomavirus (HPV), and H1N1 influenza vaccinations [1].

In Portugal, the Pfizer-BioNTech (Pfizer Inc., New York, NY; BioNTech SE, Mainz, Germany) and Moderna (Moderna, Inc, Cambridge, MA) vaccination programs against COVID-19 are now underway; even though various adverse events related to these vaccines have been reported so far, the appearance of cervical adenopathies has not been one of them [2,3]. However, Ipsilateral axillary and supraclavicular lymphadenopathies to the inoculated upper limb have been described [2,3].

## Case presentation

**Figure 1:**
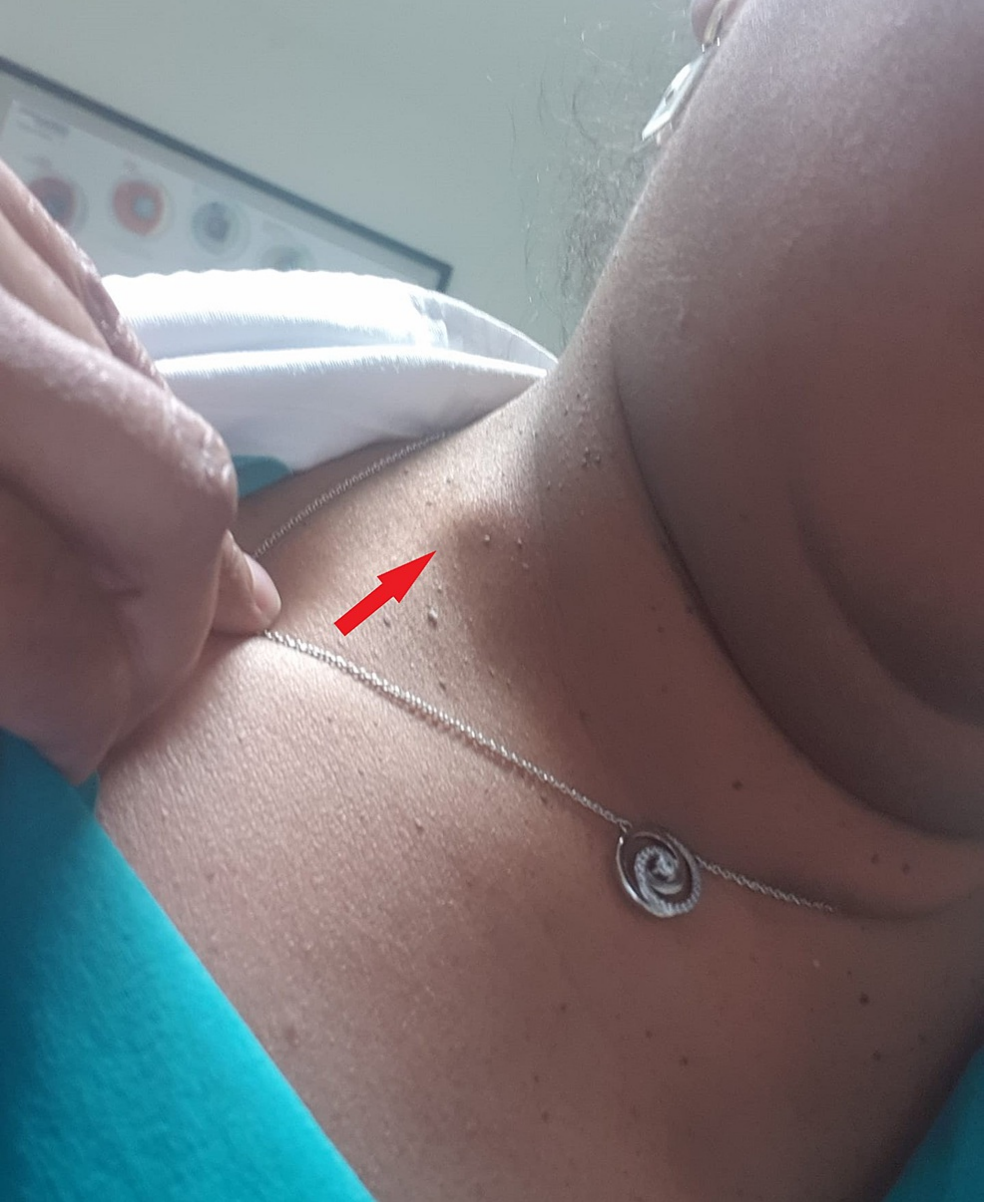
Right cervical lymphadenopathy (red arrow)

A 48-year-old female patient attended a general surgery consultation for painless lymphadenopathy of about 11 mm that had recently appeared and was located on the posterior edge of the right sternocleidomastoid muscle in its lower third (Figure 1). She reported no constitutional symptoms. The anamnesis highlighted COVID-19 vaccination with the Pfizer-BioNTech vaccine received on January 5, 2021 (two weeks before the onset of lymphadenopathy), the patient’s usual contraceptive medication, Mercilon®, and a family history of breast cancer (maternal aunt and mother).

**Figure 2:**
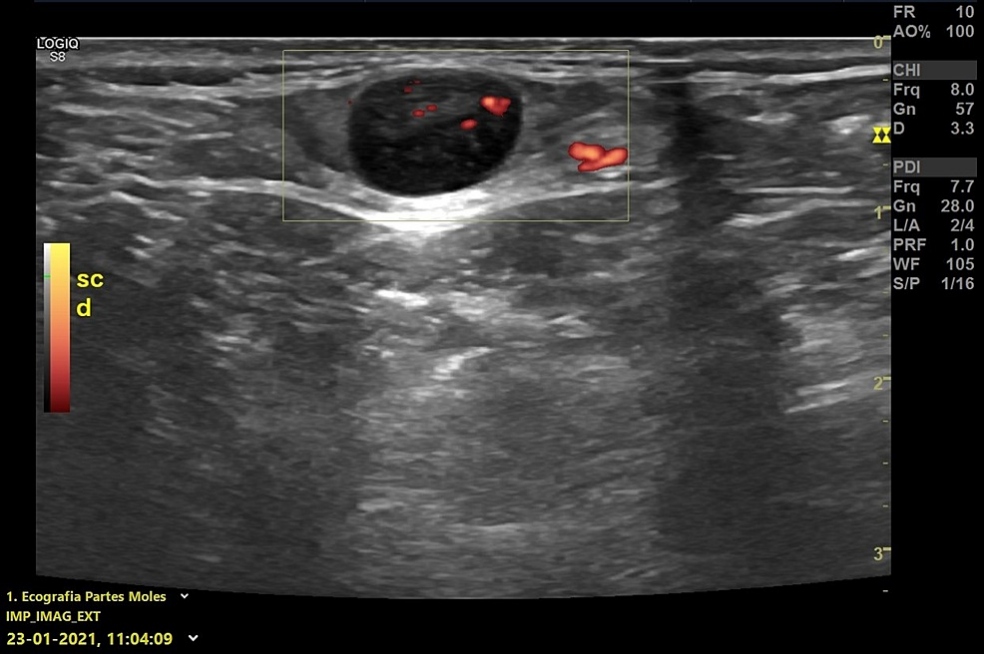
Ultrasonography - January 23, 2021 The image shows lymph node with an increase in echogenicity and in the sphericity index, without a defined hilum and 10.56 × 7.39 mm in size

Breast ultrasonography and mammography screening from December 2020 had shown no suspicious lesions. The patient underwent a cervical ultrasound on January 23, 2021, which revealed a lymph node with reactive characteristics and 10.56 × 7.39 mm in size (Figure 2). A blood test did not reveal lymphocytosis and showed several antiviral antibodies (Table 1).

**Table 1:** Laboratory results – January 23, 2021 CRP: C-reactive protein; CEA: carcinoembryonic antigen; CA 19-9: cancer antigen 19-9; CMV: cytomegalovirus; IgM: immunoglobulin M; VCA: viral capsid antigen; EBNA; Epstein-Barr virus nuclear antigen; SARS-CoV-2: severe acute respiratory syndrome coronavirus 2

Analysis	Result	Unit	Reference values
Lymphocytes	1.65	%	1.0–4.5
Immature granulocytes	0.01	%	0.2–1.0
Globular sedimentation speed VS – (1ª hour)	16	mm	0–12
CRP	5.3	mg/L	<5.0
CEA	<1.73	Ng/mL	0.0–5.0
CA 19-9	7.9	U/mL	<37
CA 15-3	17	U/mL	<31
CA 125	11	U/mL	<35
Anti-CMV IgM antibody	0.25, negative	Index	
Anti-CMV IgG antibody	160, positive	UA/mL	
Epstein-Barr VCA IgG	82.01, reactive	Index	
EBNA IgG	8.0, reactive	Index	
Epstein-Barr VCA IgM	0.05, non-reactive	Index	
Anti-SARS-CoV-2 IgG antibody (anti-nucleocapsid)	0.0, negative	Index	

The patient was inoculated with the second vaccine dose on January 26, 2021, which was administered 21 days after the first dose, following the manufacturer’s recommendation. The lymphadenopathy increased slightly in volume but remained painless.

**Figure 3:**
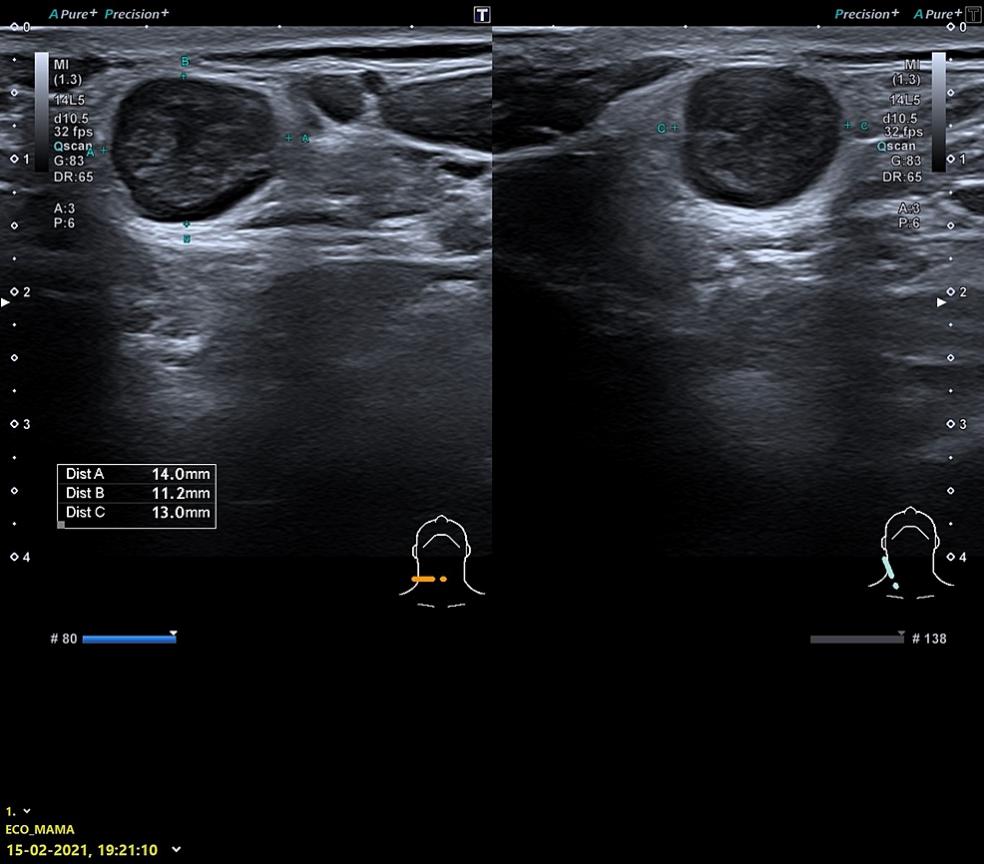
Cervical ultrasonography - February 15, 2021 The image shows one of the adenopathies with increased sphericity index, hypoechogenic, without defined hilum, and 14 x 11 mm in size

The patient underwent a neck ultrasound on February 15, 2021, which showed three adenopathies with an increase in echogenicity and in the sphericity index, without a defined hilum, close to each other, and in the right accessory spinal chain. The largest adenopathy was 14 × 11 mm in size, and the other two were 6.5 x 5.4 mm and 4.8 x 4.7 mm, respectively. Located in the transition between the laterocervical region and the homolateral supraclavicular pit, another ganglion formation was observed, which was oval in shape, also without a clearly defined hilum, and with a size of 10 × 5 mm (Figure 3). No other changes in the cervical, mediastinal, or abdominal ganglion chains were observed.

**Figure 4:**
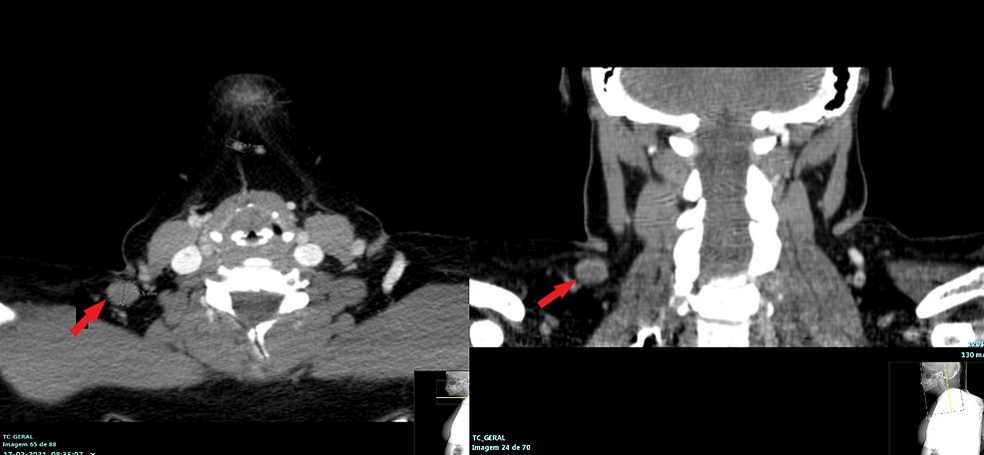
Neck CT scan - February 17, 2021 The images show a cervical ganglion of 13.7 × 13.5 mm and two adjacent others with 5 and 6 mm in size (red arrow) CT: computed tomography

A neck CT scan was performed on February 17, 2021, with the following findings: lateral cervical adenopathies on the right; a cervical ganglion of 13.7 x 13.5 mm and two others adjacent with 6.1 x 5.8 and 5.6 x 5.3 mm in size; and slight asymmetry of the pyriform sinuses with smaller amplitude of the right (Figure 4).

**Figure 5:**
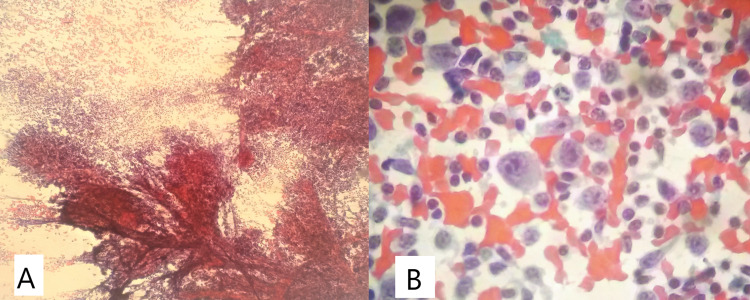
Cytology of the lymph node (hematoxylin and eosin) A: 40x; B: 400x

The patient was observed by an otolaryngologist. The ENT surgeon ruled out pharyngolaryngeal lesions. A fine-needle aspiration of the right cervical lymphadenopathy was performed on February 19, 2021: Reed-Sternberg cells or malignant epithelial neoplastic cells were not identified. The conclusion was that it showed atypical lymphoid cytology (Figure 5). Excision was suggested for better evaluation.

The patient underwent an excisional biopsy of the lymph node at right level V on March 3, 2021.

**Figure 6:**
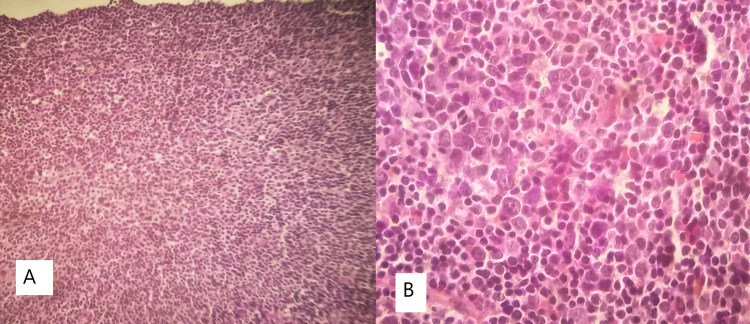
Histology of a lymph node section (hematoxylin and eosin) A: 40x; B: 400x

The histology showed reactive follicular hyperplasia. The complementary immunohistochemical evaluation was performed using the following antisera: CD3, CD5, CD10, CD20, CD23, BCL-2, BCL-6, Cyclin D1, and MUM1 (Figure 6).

Taking into account the previous cytological study, a sample was sent for consultation to a pathologist at another hospital, who agreed with the diagnosis proposed above.

## Discussion

Cervical lymphadenopathies can be a cause for serious medical concern. Although it is most commonly caused by infections, a neoplastic origin cannot be ruled out. In individuals over 50 years of age, the presence of palpable cervical adenopathy is mostly associated with malignancy, which may constitute a metastasis of a neoplasm of the airway or the digestive tract or a sign of lymphoproliferative disease.

Prior to the rolling out of COVID-19 vaccinations, immunizations were considered a rare cause of benign reactive axillary lymphadenopathy. Influenza, measles, smallpox, anthrax, and BCI vaccines have all been implicated in occasional axillary lymphadenopathy [1]. However, data from COVID-19 vaccine clinical trials suggest that the first two FDA-approved COVID-19 vaccines, the Pfizer-BioNTech and Moderna COVID-19 vaccines, which are based on the novel messenger ribonucleic acid (mRNA) technology, are highly immunogenic, with a large number of patients reporting both local and systemic reactions compared to other routine vaccines [4]. In addition, the early clinical experience of breast radiologists suggests that the two approved COVID-19 vaccines have caused numerous cases of unilateral axillary lymphadenopathy, which have been observed on breast imaging, including MRI [5,6].

The phase III randomized placebo-controlled clinical trial of the Pfizer mRNA vaccine and the Moderna trials noted a lower rate of post-vaccination lymphadenopathy and offered limited data on the time course of symptomatic lymphadenopathy [2,3]. The Pfizer trial reported unsolicited adverse events as follows: "Lymphadenopathy occurred in the arm and neck region and was reported within two to four days after vaccination. The average duration of lymphadenopathy was approximately 10 days" [3].

The European Medicines Agency has published details related to adverse reactions in clinical studies of various COVID-19 vaccines, and axillary lymphadenopathies have been documented as ranging from ≥1/1000 to <1/100 for the Pfizer-BioNTech and the AstraZeneca vaccines and much more common (≥1/10) for the Moderna vaccine [7,8]. In the few studies that have reported the development of axillary and supraclavicular lymphadenopathies ipsilateral to the limb inoculated with the COVID-19 vaccine, the average interval between inoculation with the first vaccine dose and the appearance of axillary lymphadenopathy has been reported to be 10 days (range: 2-29 days) [9].

In our clinical case, lymphadenopathy appeared at 15 days, and its structure was altered after the second dose of the vaccine. Given the patient’s family history of breast cancer, a thorough study was conducted. The ganglion resumed its form about four weeks after the second dose of the vaccine. Prior knowledge of possible adverse reactions to the COVID-19 vaccine would have mitigated the anxiety levels of the patient and the attending physician, thereby avoiding the need for the excisional biopsy.

## Conclusions

With the large-scale rollout of COVID-19 vaccination, it is critical for physicians to consider the effects of vaccination as a possible cause in the differential diagnosis of axillary, supraclavicular, or cervical lymphadenopathies. Since COVID-19 vaccines will soon be available to a larger patient population, radiologists should be familiar with the imaging features of COVID-19 vaccine-induced hyperplastic adenopathy and its inclusion in the differential diagnosis for unilateral cervical adenopathy.

We recommend that radiologists, when diagnosing any case of lymphadenopathy, perform a reassessment 4-12 weeks after the second COVID-19 vaccination dose so that diagnostic biopsies, which impose a heavy financial burden on the healthcare system, cause the risk of morbidity, and result in much patient anxiety, are avoided.
